# Fluoroscopy-guided, sheath-assisted biopsy for indeterminate intraductal biliary masses

**DOI:** 10.1055/a-2777-6802

**Published:** 2026-01-28

**Authors:** Katarzyna M. Pawlak, Jakub Juźwiak, Marek Brzosko, Jacek Piątkowski, Marek Jackowski, Mateusz Jagielski

**Affiliations:** 1 Department and Clinic of General, Gastroenterological and Oncological Surgery, Collegium Medicum, Nicolaus Copernicus University, Toruń, Poland; 2 Endoscopy Unit, Hospital of The Ministry of Interior and Administration, Szczecin, Poland; 3 Clinic of Internal Medicine, Rheumatology, Diabetology, Geriatrics, and Clinical Immunology with the Gastroenterology Department, Police, Poland

Endoscopic retrograde cholangiopancreatography (ERCP) with biliary biopsy is an established diagnostic method for cholangiocarcinoma; however, its sensitivity remains relatively low, ranging between 40 and 60% with forceps biopsies 1 . In contrast, peroral cholangioscopy with cholangioscopy-directed biopsies demonstrates higher diagnostic performance, with a pooled sensitivity of 71.9% and a specificity of 99.1% 2 . Nevertheless, due to high costs and variability in reimbursement across countries, access to cholangioscopy may be limited. In such cases, fluoroscopy-guided, sheath-supported biopsy can serve as a useful alternative.

We applied this technique in three patients with intraductal masses located in the hilar, mid, and distal bile duct, all of whom had previously undergone negative biopsies (brush cytology, blind biopsy forceps, and bile cytology). During ERCP, fluoroscopy-guided and sheet-supported biopsies were performed. The key procedural step was to localize the level of the mass on cholangiographic images ( Fig. 1 ). A stent introduction system, without a preloaded stent, was advanced over the guidewire beyond the level of the mass ( Fig. 2 ). After removing the wire, pediatric forceps were introduced through the sheath and advanced slightly beyond the introducer and opened. The entire system was then gently withdrawn under fluoroscopic guidance to target the lesion, allowing multiple biopsies to be obtained ( Fig. 3 ). Throughout the entire procedure, the introducer sheet was kept in the bile duct, providing a stable position and desired access. In the conclusion of each procedure, plastic stents were placed via the same introducer ( Video 1 ). All procedures were uneventful.

**Fig. 1:**
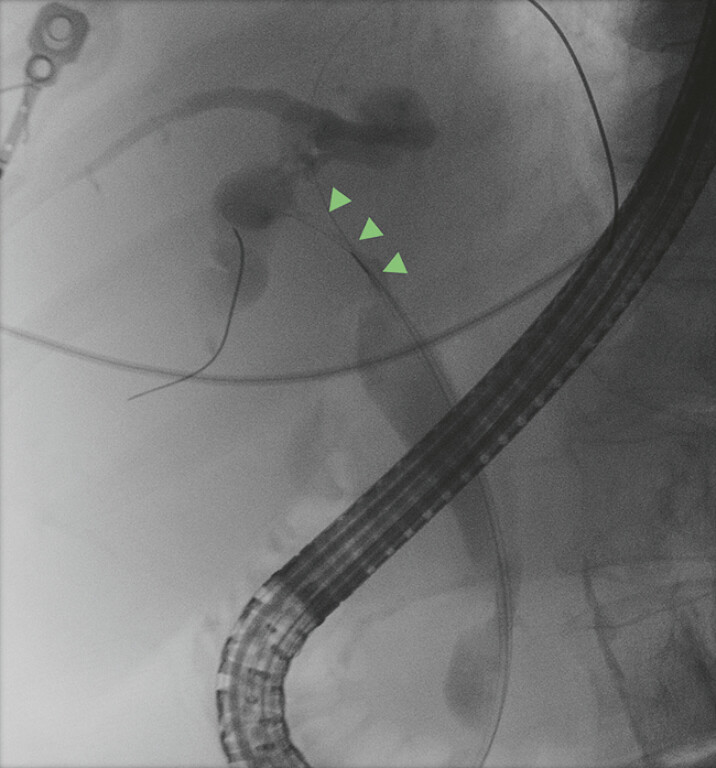
A fluoroscopic image: The mass in the hilum was visualized.

**Fig. 2:**
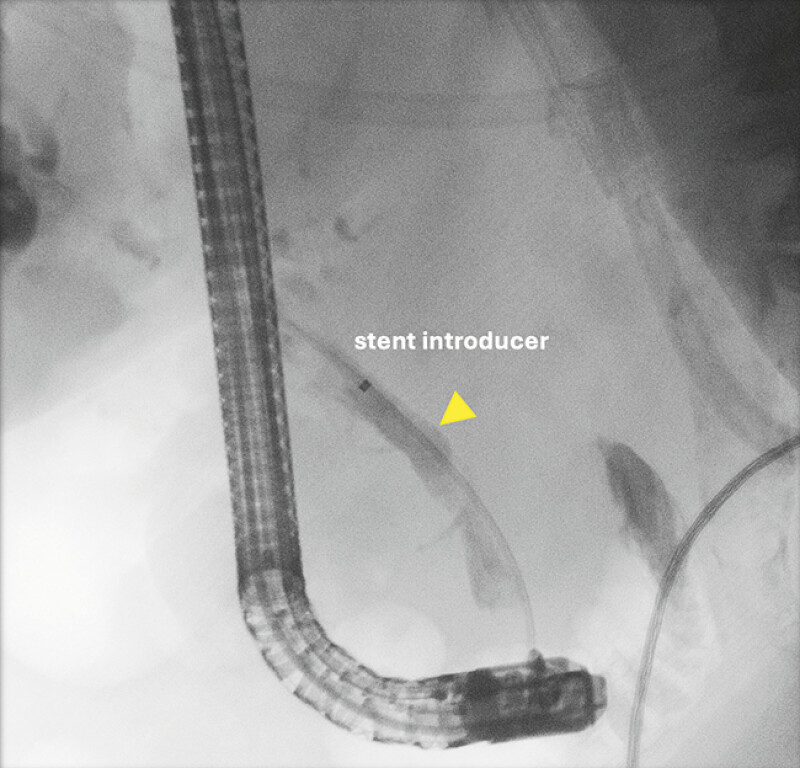
A stent introducer sheath was advanced over the wire just above the level of a mass.

**Fig. 3:**
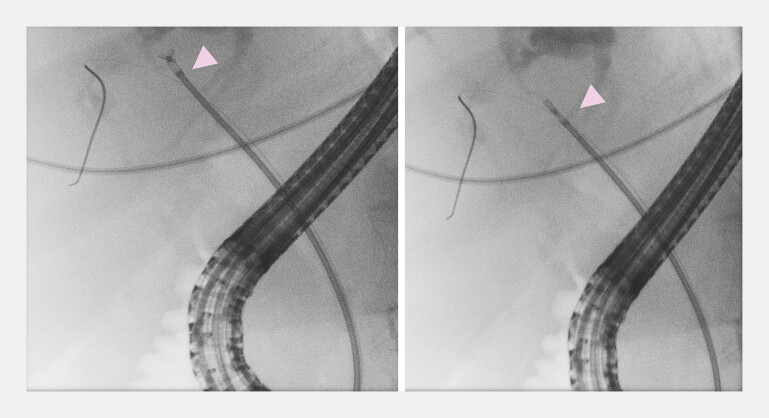
Pediatric biopsy forceps were advanced through the stent introducer, opened and pulled back to the level of mass. Multiple biopsies were obtained. The plastic stent was placed over the same introducer.

Fluoroscopy-guided, sheet-supported biopsy of non-diagnosed intraductal biliary masses.Video 1

Histopathological examination revealed intraductal papillary neoplasm of the bile ducts with low- and high-grade dysplasia in one case and adenocarcinoma in two cases.

Fluoroscopy-guided, sheath-assisted biopsy represents a simple and accessible diagnostic option for intraductal biliary masses, particularly in settings where cholangioscopy is unavailable, providing diagnostic support when other modalities prove insufficient.

Endoscopy_UCTN_Code_TTT_1AR_2AK

## References

[1] Kamp EJCA Dinjens WNM Doukas M Optimal tissue sampling during ERCP and emerging molecular techniques for the differentiation of benign and malignant biliary strictures Therap Adv Gastroenterol 2021 14 17562848211002023 10.1177/17562848211002023 PMC8053835 33948111

[2] BadshahMBVanar V Kandula M Peroral cholangioscopy with cholangioscopy-directed biopsies in the diagnosis of biliary malignancies: a systemic review and meta-analysis Eur J Gastroenterol Hepatol 2019 31 935 940 30896553 10.1097/MEG.0000000000001402

